# Human leukocyte antigen class II quantification by targeted mass spectrometry in dendritic-like cell lines and monocyte-derived dendritic cells

**DOI:** 10.1038/s41598-020-77024-y

**Published:** 2021-01-13

**Authors:** A. Casasola-LaMacchia, M. S. Ritorto, R. J. Seward, N. Ahyi-Amendah, A. Ciarla, T. P. Hickling, H. Neubert

**Affiliations:** 1 Quantitative Biomarkers and Biomeasures, BioMedicine Design, Pfizer, Inc., Andover, MA USA; 2 Immunogenicity Sciences, BioMedicine Design, Pfizer, Inc., Andover, MA USA

**Keywords:** Immunology, Peptides, Proteins, Proteomics

## Abstract

The major histocompatibility complex II (HLA-II) facilitates the presentation of antigen-derived peptides to CD4+ T-cells. Antigen presentation is not only affected by peptide processing and intracellular trafficking, but also by mechanisms that govern HLA-II abundance such as gene expression, biosynthesis and degradation. Herein we describe a mass spectrometry (MS) based HLA-II-protein quantification method, applied to dendritic-like cells (KG-1 and MUTZ-3) and human monocyte-derived dendritic cells (DCs). This method monitors the proteotypic peptides VEHWGLD*K*PLLK, VEHWGLD*Q*PLLK and VEHWGLD*E*PLLK, mapping to the α-chains HLA-DQA1, -DPA1 and -DRA1/DQA2, respectively. Total HLA-II was detected at 176 and 248 fmol per million unstimulated KG-1 and MUTZ-3 cells, respectively. In contrast, TNF- and LPS-induced MUTZ-3 cells showed a 50- and 200-fold increase, respectively, of total α-chain as measured by MS. HLA-II protein levels in unstimulated DCs varied significantly between donors ranging from ~ 4 to ~ 50 pmol per million DCs. Cell surface HLA-DR levels detected by flow cytometry increased 2- to 3-fold after DC activation with lipopolysaccharide (LPS), in contrast to a decrease or no change in total HLA α-chain as determined by MS. HLA-DRA1 was detected as the predominant variant, representing > 90% of total α-chain, followed by DPA1 and DQA1 at 3–7% and ≤ 1%, respectively.

## Introduction

Specialized antigen presenting cells, such as dendritic cells (DCs), play a fundamental role in the adaptive immune response by presenting antigen-derived peptides to T-cells. These peptides are displayed at the cell surface by the HLA-II complex. According to the linear model of DC maturation, immature DCs are functionally poor activators of T cells but have a high capacity for internalizing antigenic compounds through active endocytosis^1–4^. This development state correlates with low levels of HLA-II at the cell surface. At the onset of maturation, HLA-II is redistributed to the plasma membrane and T-cell stimulating activity of DCs increases significantly^1–7^. HLA-II is composed of two ~ 26 kDa-transmembrane subunits, the α- and β-chains. Both chains have similar structures consisting of a cytoplasmic tail and two extracellular domains, which combine their distal domains to form a single peptide binding groove.

HLA-II is encoded in the human MHC locus by three major gene families: HLA-DR, -DP and -DQ, all of which are concomitantly expressed with their respective α- and β-chains. After a finely co-regulated transcription, α- and β-chain mRNAs are translocated in a coordinated manner to the cytoplasm for translation^8–16^. HLA-II heterodimers assemble in the endoplasmic reticulum to subsequently associate with the class-II chaperone CD74, which subsequently is partially digested, leaving the residual class-II-associated invariant chain peptide (CLIP) bound to the binding pocket of the HLA-II. CLIP peptides are exchanged for high affinity peptides derived from degraded proteins^17,18^. Peptide loaded-HLA-II subsequently translocates from late endosomes to the plasma membrane, where it is stabilized, a functional characteristic of DC maturation. CLIP- and antigenic peptide-complexes with HLA-II can also be internalized to antigenic processing compartments via clathrin-dependent or -independent endocytosis^1,8,18–22^. Therefore, total HLA-II levels are governed by the interplay between transcriptional activity, protein synthesis and the rate of HLA-II internalization, recycling and degradation^23–26^. However, the dynamics of each of these processes likely varies in populations of phenotypically diverse human DCs.

Furthermore, many alleles and polymorphisms exist not only in HLA-II α and β chains, but also in the multiple molecular regulators identified so far at the transcriptional and post-transcriptional level^10,16,27–31^. This adds complexity to the mechanisms underlining HLA-II abundance, which explains in part, the wide variability of peptide presentation specificity by DCs. Moreover, polymorphisms in the non-coding regulating sequences 5′ and 3′ untranslated-regions (UTRs) as well as the promoters of HLA-DQ genes are involved in upregulation of HLA-II expression such as HLA-DQB1*03:01 and *02:01^10,31^, which likely contributes to differences in peptide presentation. Consistently, multiple allelic variants are linked to a higher risk of specific human disorders such as type 1 diabetes (specific HLA-DR-DQ haplotypes) and graft versus host disease (GVHD) after hematopoietic transplantation as well as high incidence of viral infections^32,33^. While accumulating evidence suggests these correlations between specific polymorphisms and HLA alleles to states of disease at the genetic level, the posttranscriptional HLA-II variation and its multifactorial regulation in terms of abundance and functional significance remains largely unknown.

While HLA-II mRNA has been quantified and HLA-II protein detected by western blot, immunohistochemistry and flow cytometry, more accurate methods for quantification of HLA-II protein have not yet been demonstrated. To this end, we developed a quantitative assay for total HLA-II-alpha chains based on HLA-II immunopurification, trypsin digestion and liquid chromatography tandem mass spectrometry (LC–MS/MS) using parallel reaction monitoring (PRM) data acquisition in a high-resolution mass spectrometer. LC–MS/MS analysis of the HLA-II tryptic peptides confers both specific identification and sensitive quantification. In the current work, the targeted peptides map to HLA-DRA1/DQA2, -DPA1 and -DQA1, differing only in one amino acid: VEHWGLD*E*PLLK, VEHWGLD*Q*PLLK and VEHWGLD*K*PLLK (Supplementary Fig. S1). To establish a baseline for the detection of HLA-II in an in vitro cell model, we initially quantified the HLA-II-alpha chains in the acute myeloid leukemia cell line KG-1, a consistent source of HLA-II protein. Subsequently, we demonstrated the quantification of HLA-II from the dendritic-like cell model MUTZ-3^34–41^, as well as a population of myeloid DCs (CD11c+) derived from human blood, both of which can be differentiated and activated by proinflammatory stimuli. Furthermore, these CD14+ monocyte-derived dendritic cells were activated with LPS to examine HLA-DR at the cell surface as well as total HLAII alpha chains.

## Materials and methods

### Cell culture

KG-1 (CCL-246, KG-1 AML, Lot 63011477) and MUTZ-3 (ACC 295) cell lines were purchased from ATCC (Rockville, USA) and DSMZ (Braunschweig, Germany), respectively. Both cell lines were cultured in a humidified incubator at 37 °C with 5% CO_2_ as described^35–41^. Briefly, KG-1 cells were cultured in RPMI 1640 Medium (RPMI; Gibco, Thermo Fisher Scientific, MA, USA) supplemented with 20% (v/v) Fetal Bovine Serum (FBS; Hyclone, Logan, USA) and penicillin/streptomycin (100 U/mL); MUTZ-3 cells were cultured with α-Minimum Essential Medium (α-MEM; Gibco, Thermo Fisher Scientific) supplemented with 20% (v/v) FBS, 100 U/mL penicillin/streptomycin and 25 IU/mL GM-CSF (R&D systems, Minneapolis, USA). MUTZ-3 maturation was stimulated as previously described^34,38,41^. Briefly, 2–4 × 10^5^ cells/mL were seeded in complete media with 50 ng/mL GM-CSF and 20 ng/mL Interleukin-4 (IL-4, R&D systems) for 7 days. For mature MUTZ-3 activation 12 ng/mL TNF (R&D Systems) or 1 μg/ml of LPS (Sigma-Aldrich) were added independently at the end of day 5 and then cells were harvested at day 7 and stored at − 80 °C.

### Monocyte-derived DCs

To generate DCs, CD14+ monocytes were isolated from PBMCs collected with informed consent from six healthy donors (D1095, D1995, D18092, D1237, D1265, D1765, STEMCELL Technologies, Vancouver, BC, Canada). PBMCs from half-leukopaks were isolated by Ficoll (GE Health Sciences) density separation and CD14+ monocytes were isolated by magnetic bead-based cell separation (MACS, Miltenyi Biotec, MA, USA), using microbead-conjugated anti-CD14 antibodies and magnetic cell separation columns (Miltenyi Biotec) according to the manufacturer protocol. CD14+ monocytes were differentiated into DCs using a modification of a previously published protocol^42–45^. Briefly, monocytes were cultured at a density of 5 × 10^5^ cells/mL in RPMI medium supplemented with 10% (v/v) FBS, GM-CSF (100 ng/mL) and IL-4 (17 ng/mL) for 6 days. To determine the LPS concentration for DC activation, independent sets of monocytic-DCs derived from four healthy human donors were activated with six increasing concentrations of LPS (0, 0.3, 1, 10, 32, 250 and 1000 ng/mL) on Day 5 as previously described ^46^. This titration led to the selection of 32 ng/ml of LPS for stimulation of experimental monocytic DCs due to the consistent performance and activation response. On day 6 cells were harvested 22 h post LPS addition, washed with PBS three times, counted and stored at − 80 °C.

### Flow cytometry

DCs were harvested pre- and post-LPS stimulation and cell surface-associated HLA-DR (csHLADR) was detected by flow cytometry after staining with an anti-HLA-DR L243 antibody in non-permeabilized DCs. Briefly, cell pellets were washed with FACS staining buffer+ BSA (BD Pharmingen, 554,657) and stained for 30 min with the following conjugated antibodies: Alexa 488-HLA-DR (Clone L243; Biolegend, 307,620 B241328), PE-CD86 (Clone IT2.2; Biolegend, 305,406 B210795), BV421-CD11c (Clone 3.9; Biolegend, 301,628 B238001), APC-CD40 (Clone 5C3; Biolegend, 334,310 B219298) and live/dead fixable near-IR dead cell stain (Lifetech, L34976 1,868,118). Cells were washed three times with staining buffer and cell surface stain was analyzed with a BD Fortessa flow cytometer using BD FACS DIVA software. Further analyses were carried out using FlowJo software V.10.

### Cell lysis, HLA-II-immunoprecipitation and proteolytic digestion

Cell lines and DCs were processed in an identical manner as described below. To obtain whole-cell lysates, pelleted cells were thawed and resuspended in 1 mL of cold lysis buffer containing 20 mM Tris, pH 8.0, 150 mM NaCl, 1% (v/v) CHAPS+ 1 mM PMSF (Sigma, A3428-10MG), 5 μg/mL Aprotinin (Sigma, A3428-10MG), 10 μg/mL Pepstatin A (Calbiochem, 516,481-5MG) and 10 μg/mL Leupeptin (Sigma, L5793-5MG). Lysates were incubated for 1 h with end-to-end rotation at 4 °C then centrifuged at 2500 RCF for 5 min to recover the supernatant. In order to establish the linearity of signal detection in a titration experiment, serial dilutions of 200 μL whole lysate per cell type and condition were performed in duplicate in a 96-well plate format (LoBind deep-well plate, Eppendorf) to obtain samples corresponding to 100,000, 50,000 25,000, 12,500, 6250, and 3125 cells as well as blanks.

Immunoprecipitation (IP) of total HLA-II protein was carried out in each sample (cell lysates equivalent to 100,000, 50,000 25,000, 12,500, 6250, and 3125 cells) by adding 10 μg of anti-panHLA-II antibody (ATCC HB-145 hybridoma, anti-HLA-DP/DQ/DR antibody), which was biotinylated using EZ-Link Sulfo-NHS-LC-Biotin (Thermo Fisher Scientific, 21,327). Antibody incubation proceeded overnight at 4 °C while shaking at 600 rpm. 50 μl of streptavidin magnetic beads (T1 MyOne, Thermo Fisher Scientific) were washed once with 1× PBS+ 0.1% Tween (PBST) and twice with 1× PBS prior to being added to each IP sample in a 96-well LoBind plate and incubated for 1.5 h while shaking at 1200 rpm at RT. To examine the completeness of HLA-II recovery during the immunoprecipitation step, a secondary IP was performed on the post-IP lysates, with selected samples, using the identical methodology described above in order to assess how much HLA-II α-chain, if any, remained in the lysate following IP.

Bead bound immunocomplexes (HLA-II-mAb-beads) were washed twice with PBST buffer followed by one wash with PBS before elution with 135 μL of 30 mM HCl/5% ACN using an automated bead handling system (KingFisher Flex, Thermo Fisher Scientific). Lyophilized stable isotope labeled (SIL) peptides (lysine ^13^C_6_,^15^N_2_) VEHWGLD*K*PLL**K**, VEHWGLD*Q*PLL**K** and VEHWGLD*E*PLL**K** (New England Peptide, Massachusetts, USA ) were reconstituted in 30% ACN, 0.1% TFA at 69.3 pmol/μL. Peptides were diluted to 69.3 fmol/μL in an equimolar mix with 5%ACN, 0.1%TFA immediately prior to use. Samples were spiked with 4.6 μL of equimolar SIL peptide mix to a final concentration of 1.6 fmol/ μL (332 fmol of each SIL-peptide per sample). Reduction was performed by adding 2 μL of 500 mM TCEP (TCEP Bond Breaker, Thermo Fisher Scientific, 77,720) and incubation for 30 min at 60 °C. Alkylation was performed by the addition of 5 μL of 150 mM iodoacetamide to each sample followed by a 30 min incubation in the dark at RT. Sequencing grade lyophilized trypsin/Lys-C mix (Promega, V5071) was reconstituted in 20 mM Tris at 200 ng/μL immediately before use. Samples were then digested with 1 μg of trypsin/Lys-C overnight at 37 °C. Each sample digest was quenched by addition of 16 μL of 1% of TFA yielding a final volume of 200 µL.

### Liquid chromatography–tandem mass spectrometry (LC–MS/MS)

Protein digests were separated with a Thermo Fisher Scientific Dionex UltiMate 3000 UHPLC system consisting of a RSLCnano system (NCS350 nano-pump and a loading-pump combined with a temperature-controlled dual 10-port valve column compartment operated at 50 °C) and a WPS-3000 RS autosampler with a 50 μL sample loop. The system was controlled by Chromeleon Xpress software (version 6.8). 28 μL of each sample was loaded onto a C18 PepMap100 trap precolumn (300 μm i.d. × 5 mm, 5 µm, 100 Å, Thermo Fisher Scientific, 160,454 ) followed by forward elution and chromatographic separation on an EASY-Spray PepMap100 RSLC C18 nanocolumn operated at 50 °C (15 cm × 75 μm, 3 μm, 100 Å, Thermo Fisher Scientific, ES800A) at a flow rate of 400 nL/min using mobile phase A (water plus 0.1% formic acid) and mobile phase B (acetonitrile plus 0.1% formic acid). The chromatographic gradient was as follows: 0–3 min (3% B); 3–18 min (3–35% B); 18–21 min (35–45% B); 21–22 min (45–95% B); 22–26 min (95% B); 26.0–26.1 min (95–3% B) and 26.1–35.1 min (3% B). A Thermo Fisher Scientific Q Exactive HF quadrupole-Orbitrap mass spectrometer equipped with an EASY-Spray source was operated in positive ion mode for parallel reaction monitoring (PRM) of VEHWGLD*K*PLLK, VEHWGLD*Q*PLLK and VEHWGLD*E*PLLK endogenous and SIL peptides. The PRM method parameters were as follows: MS2 was acquired at 30,000 resolution at m/z 200 and an isolation window at *m*/*z* 1.2; an AGC of 5e5 per target with an injection time (IT) of 100 ms. The PRM ion transitions were selected for the three endogenous and corresponding SIL-peptides according to Table 1. Each sample was analyzed by LC–MS/MS in duplicate.

**Table 1:** Precursor and fragment ion transitions and their charge states monitored by PRM for HLA α-chain quantification.

HLA-α type	Surrogate tryptic peptide	Precursor to fragment Ions	
DRA1/DQA2	VEHWGLD*E*PLLK	479.2575+++ (light)	479.2575+++ (heavy)
		E [y5] − 599.3763 +	E [y5] − 607.3905+
		E [y11] − 668.8484++	E [y11] − 672.8555++
		H [y10 ] − 604.3271++	H [y10] − 608.3342++
DPA1	VEHWGLD*Q*PLLK	478.9295+++ (light)	481.6009+++ (heavy)
		Q [y5] − 598.3923+	Q [y5] − 606.4065+
		E [y11] − 668.3564++	E [y11] − 672.3635++
		H [y10] − 603.8351++	[H [y10] − 607.8422++
		W [y9] − 535.3057++	W [y9] − 539.3128++
DQA1	VEHWGLD*K*PLLK	478.9416+++ (light)	481.6130+++(heavy)
		K [y5] − 598.4287 +	K [y5] − 606.4429 +
		E [y11] − 668.3746++	E [y11] − 672.3817++
		H [y10] − 603.8533++	H [y10] − 607.8604++
		W [y9] − 535.3239++	W [y9] − 539.3310++

### Data processing

To determine the total HLA-II levels, the peak area ratios of endogenous peptides to SIL reference peptides were calculated and multiplied by the total amount of reference SIL-peptide spiked per sample (332 fmol). Typically, lysates corresponding to 100,000, 50,000, 25,000, 12,500, 6250, 3125 and 0 cells were analyzed. Each quantification result was based on a total of six measurements as follows. Duplicate samples each with 100,000, 50,000 and 25,000 cells were used to derive the HLA-II α-chain level in each cell type or donor as well as to confirm linearity of HLA-II α-chain detection. Total HLA α-chain levels for each measurement were then normalized to 1 × 10^6^ cells and averaged across the three different starting cell numbers to obtain the final amount of HLA-DRA1/DQA2, -DPA1 and -DQA1 (fmol/1 × 10^6^). Of note, data from the lower cell numbers (12,500 6250 and 3125 cells) expectedly decreased in signal intensity and for some cell types resulted in low signal intensity unreliable for further data analysis. Thus, these lower cell numbers across all conditions were not considered in the final quantification result.

### Statistical analysis

To evaluate statistical significance of differences in the amount of csHLA-II and totalHLA-II detected by flow cytometry and LC–MS/MS, *p* values were obtained with two-tailed Student's *t* Test analyses.

## Results and discussion

**Figure 1:**
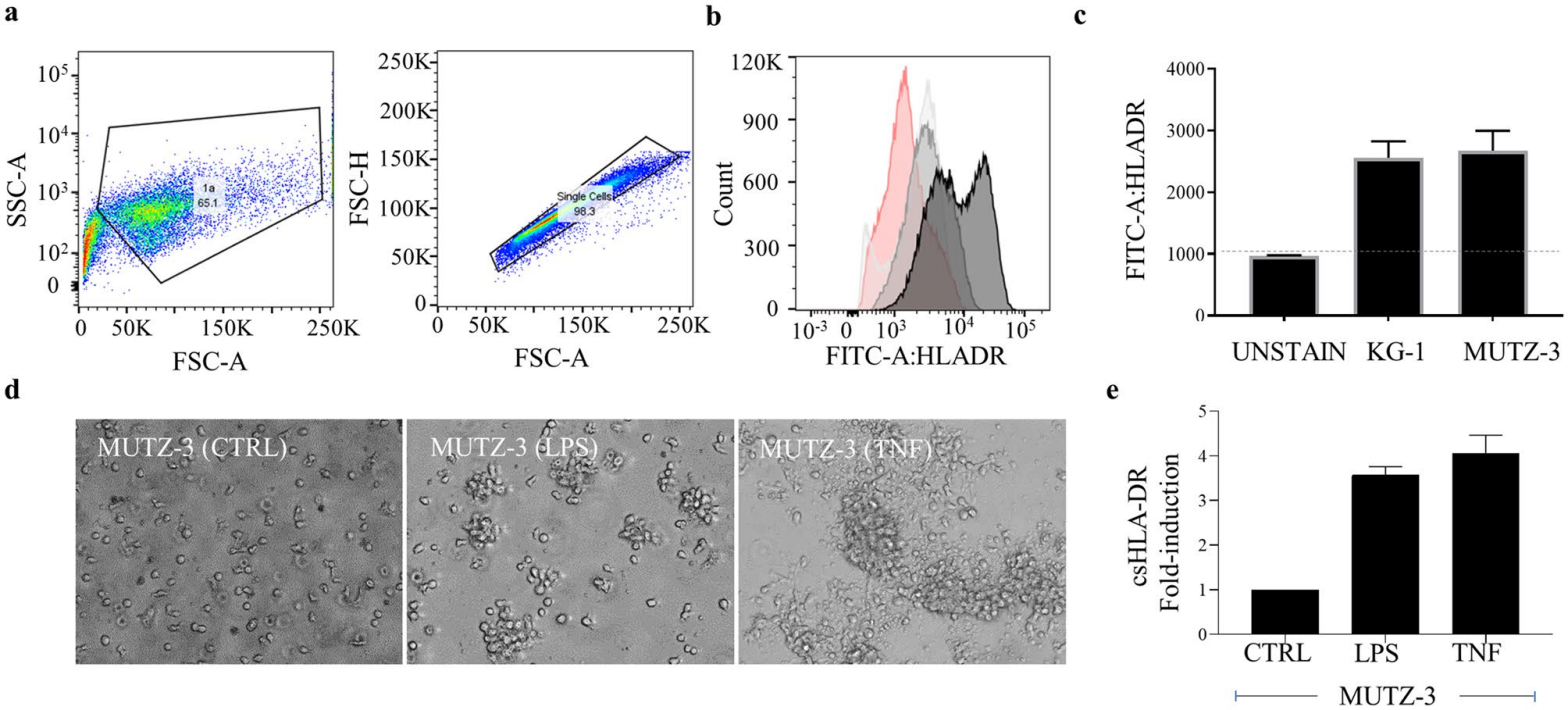
Proinflammatory stimulants LPS and TNF induce HLA-DR at the cell surface (csHLA-DR) in dendritic-like cell model. (**a**) KG-1 dendritic-like cell populations analyzed by FACS gated by SSC-A and FSC-A as well as FSC-H and FSC-A, representative plots. (**b**) csHLA-DR in KG-1 non-stained control (red), KG-1 (light gray), MUTZ-3 (dark gray) and stimulated MUTZ-33 (black). (**c**) Median fluorescence intensity (MFI) FITC-A:HLADR signal of KG-1 and MUTZ-3 cell lines normalized to unstained control (n = 3), each bar represents the mean and the standard deviation (SD). (**d**) Morphology of unstimulated and stimulated MUTZ-3 cells by light microcopy (40x). Left to right, unstimulated MUTZ-33 cells (control, CTRL), MUTZ-3 treated with LPS or TNF at 1 µg/mL and 100 ng/mL, respectively. (**e**) csHLA-DR fold induction measured by flow cytometry in stimulation conditions normalized to unstimulated control (n = 3), each bar represents the mean and the SD.

*HLA-DR analysis on the cell surface of KG-1 and MUTZ-3.* To detect cell surface associated-HLA-DR (csHLADR) by flow cytometry, the myeloid derived-cell lines KG-1 and MUTZ-3 were stained with the anti-HLA-DR antibody (L243) (Fig. 1A,B). Without stimulation, csHLADR was observed at approximately equivalent levels in both KG-1 and MUTZ-3 cell lines (Fig. 1C). MUTZ-3 cells were differentiated into DC-like cells upon incubation with low levels of GM-CSF and IL-4 and further activation with proinflammatory stimuli. Phenotypic changes were induced in MUTZ-3 cells by LPS or TNF resulting in clustered cells loosely attached to adherent counterparts and the presence of pseudopodia (Fig. 1D). MUTZ-3 cells showed a 3- to 4-fold increase in HLA-DR levels at the cell surface upon both stimuli (Fig. 1E).

### HLA-DR analysis on the cell surface of monocyte-derived DCs

**Figure 2:**
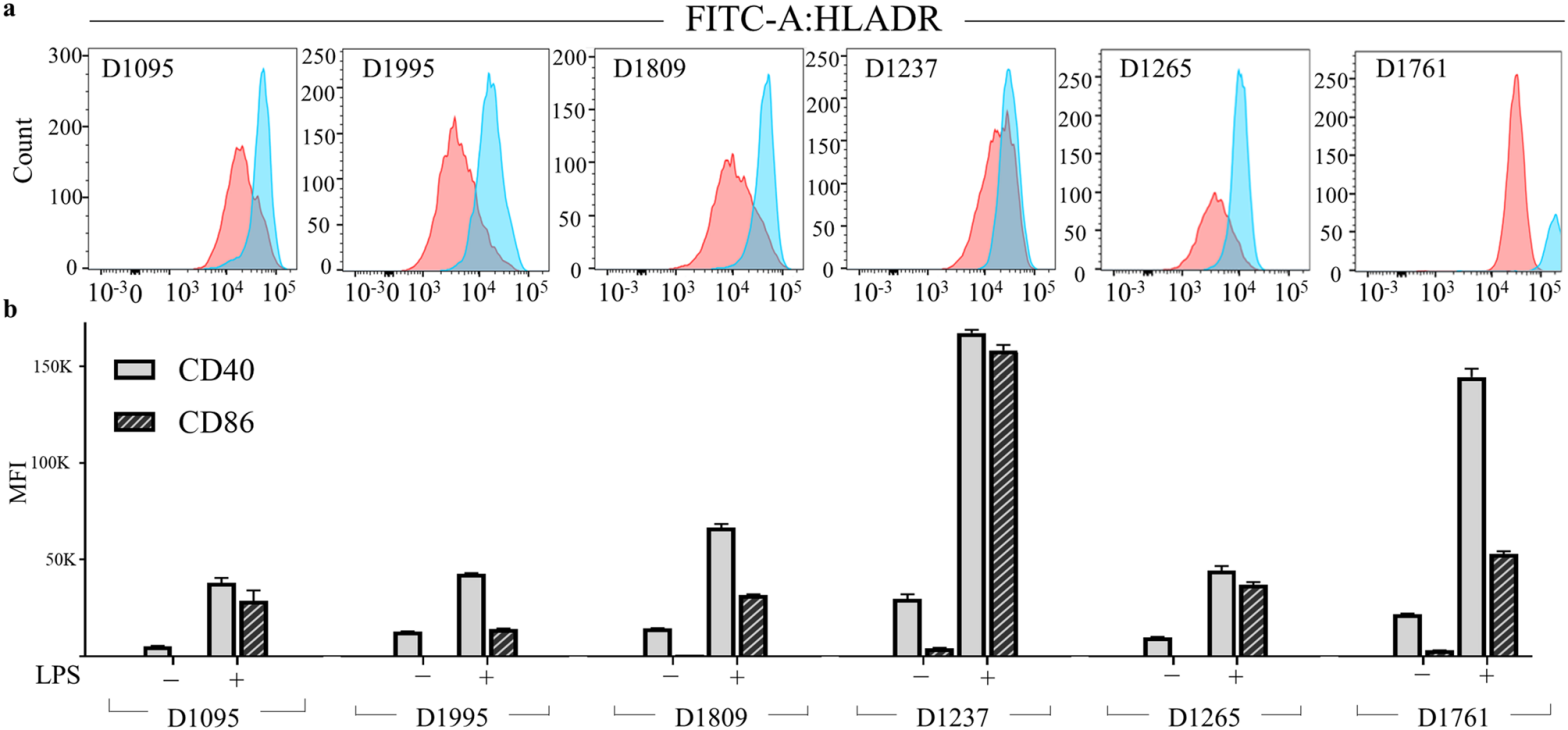
Increase of HLA-DR, CD40 and CD86 at the plasma membrane confirms DC activation upon LPS treatment. (**a**) csHLA-DR detection by flow cytometry in donor-derived monocytic DCs activated with LPS (blue) in comparison with unstimulated DCs (red), D1761 singlet analysis not available. (**b**) Activation markers CD40 (gray bars) and CD86 (striped bars) in 6 sets of donors presented as median fluorescence intensity (MFI) of APC and PE, respectively, each bar represents the mean and the SD. Statistical differences between unstimulated and LPS-treated in three replicates from (**a**) and (**b**) were evaluated with a two-tailed Student's *t* Test (*p* < 0.05 and *p* < 0.001, respectively).

We explored if the LPS-induced increase of csHLADR could also be observed in human-derived DCs. CD14+ monocytes from PBMCs from six healthy human donors with different HLA backgrounds (Table 2) were isolated and differentiated in vitro to monocytic DCs. Subsequent DC activation was performed based on LPS-titrations established in DCs derived from four independent donors (Supplementary Fig. S2). Here, HLA-DR and csHLADR abundance did not change at concentrations > 1 ng/mL of LPS when detected by western blot and flow cytometry (Supplementary Fig. S2A,B), which was also consistent with the equivalent abundance of CD40 and CD86 (Supplementary Fig. S2C,D). Levels of csHLADR increased 1.5 to fourfold in CD11c+ singlets for all LPS-induced DCs (Fig. 2A). Although csHLADP, csHLADQ and other cell surface markers were not analyzed by flow cytometry in this panel, DC-maturation upon LPS was also confirmed in all cases with the robust increase in detection of the costimulatory molecules CD40 and CD86 at the cell membrane (Fig. 2B).

**Table 2:** HLA haplotype per donor based on genotype.

Donor ID	DRB1		DRB3		DRB4	DRB5			DQB1		DpB1	
	DRB11	DRB12	DRB31	DRB32	DRB41	DRB42	DRB51	DRB52	DRB11	DRB12	DRB11	DRB12
1905	03:01:01	11:01:01							02:01:01	03:01:01	04:01:01	04:01:01
1995	11:01:01	13:02:01		03:01:01					03:01:01	06:09:01	04:01:01	05:01:01
1809	01:01:01	03:01:01							02:01:01	05:01:01	03:01:01	104:01:01
1237	01:01:01	07:01:01			01:03:01				02:02:01	05:01:01	04:02:01	14:01:01
1265	07:01:01	15:01:01			01:03:01:02N		01:01:01		03:03:02	06:02:01	02:01:02	13:01:01
1761	03:01:01	04:01:01	01:01:02		01:03:01:02				02:01:01	03:02:01	02:01:02	02:01:02

### HLA-II α-chain abundance in cell lines by western blot

**Figure 3:**
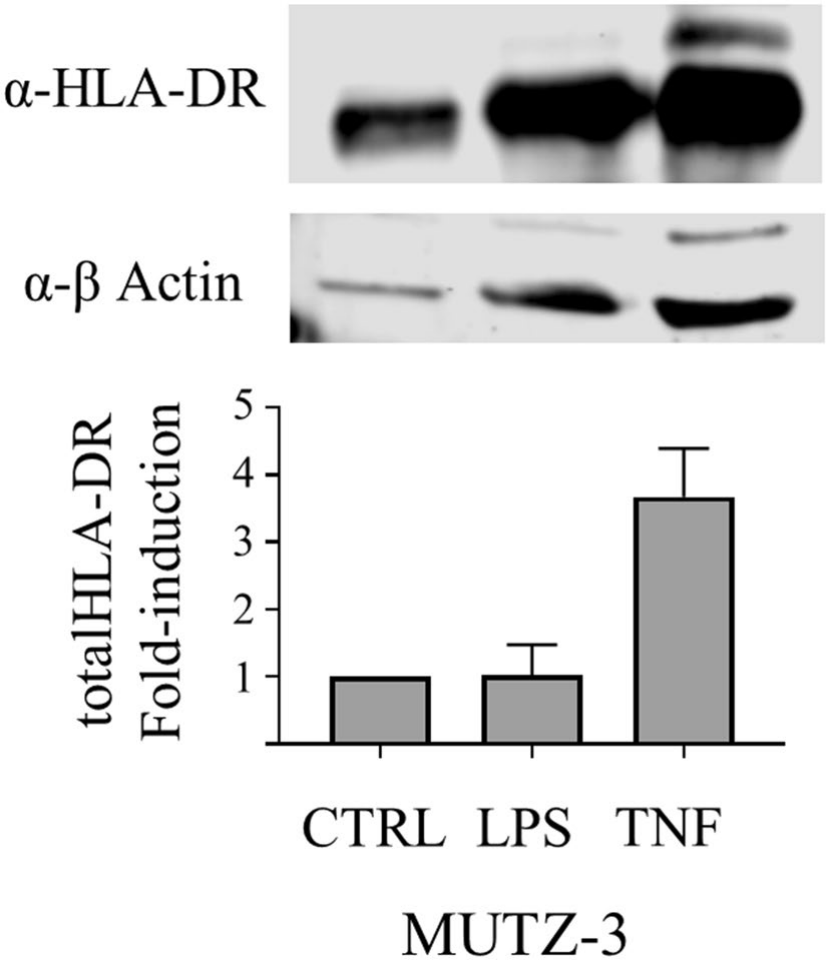
Detection of HLAII by Western Blot recapitulates increase in membrane associated-HLADR levels in MUTZ-3 cells induced to maturation. Detection of HLA-DR protein in whole lysates from MUTZ-3 cells by western blot (n = 3), each bar represents the mean and the SD. Total HLA-DR signal was normalized to the control (β-actin) and subsequently, fold induction upon LPS- and TNF-treatments was calculated based in unstimulated MUTZ-3 (CTRL).

We detected HLA-DR protein by western blot (WB, method details provided in Supporting Information, Supplementary Fig. S3) to evaluate if the increase of csHLADR in LPS-induced MUTZ-3 cells observed by flow cytometry was a consequence of an increased translocation to the plasma membrane or an increase in the total HLA-II protein amount. Total HLA-DR protein signal normalized by β-actin control increased 2– to fourfold in LPS and TNF-stimulated cells compared with undifferentiated MUTZ-3 (Fig. 3). While this data supports the increase in csHLADR in LPS-induced MUTZ-3 cells observed by flow cytometry, WB methods not only lack the ability to differentiate between HLA-DR, -DP and -DQ variants and but are also unsuitable for reliable quantification, especially in the absence of a protein standard. Further confounding WB normalization and interpretation was that actin, used as a loading control, showed an apparent increase in stimulated MUTZ-3 cells, which is consistent with the upregulation of actin expression in myeloid DCs^47–49^. These observations prompted the development of an alternative, more reliable quantification method using mass spectrometry.

### LC-MS/MS method development

**Figure 4:**
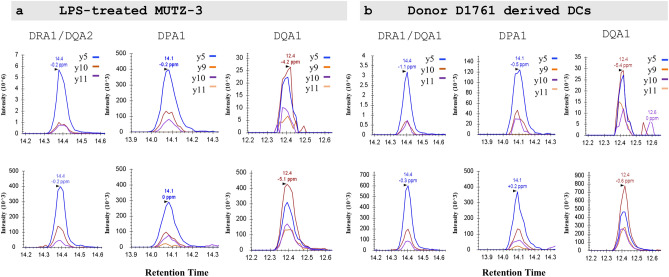
Ion chromatograms detecting endogenous (top panel) and spiked stable isotope labelled peptides (bottom panel). VEHGLD*E*PLLK (DRA/DQA2); VEHGLD*Q*PLLK (DPA1); VEHGLD*K*PLLK (DQA1) by parallel reaction monitoring (PRM) in trypsin digested lysates from (**a**) LPS-treated MUTZ-3 and (**b**) donor D1761-derived DCs. Representative signals of fragment ion transitions for each HLA α-chain detected in HLAII-immunoprecipitants equivalent to 100,000 cells for (**a**) and (**b**) are presented.

We next developed an HLA-II LC-MS/MS assay based on HLA-II-immunopurification using an anti-panHLA-II antibody (HB-145), which is frequently used in HLA-II immunopeptidomics studies^50,51^. At the amino acid-sequence level, HLA-II α-chains are less variable relative to HLA-II β-chains. We therefore targeted the HLA-II α-chain polypeptides from the three major groups of HLA variants: HLA-DRA1/DQA2, -DPA1 and -DQA1 by monitoring the proteotypic peptides VEHWGLD*E*PLLK, VEHWGLD*Q*PLLK and VEHWGLD*K*PLLK respectively. These tryptic peptides correspond to residues 190–201 in the alpha-2 domain of the HLA α-chains. Of note, quantification based on the peptide VEHWGLD*E*PLLK was reported here as HLA-DRA1, since HLA-DQA2 protein is not expressed in immune cells^47–49,52^. Despite their similarity, the three peptides and the SIL counterparts could be chromatographically separated and specifically measured by MS in PRM mode. HLA-DRA1/DQA2, -DPA and -DQA1 peptides eluted at approximately 12.4, 14.1 and 14.4 min, respectively (Fig. 4). As expected, the relative intensity of fragment ions was similar for both endogenous (light) and reference SIL-peptide (heavy) for the three target sequences. The averaged peak area ratio from duplicates of each sample corresponding to 25,000, 50,000 and 100,000 cells showed the expected increase of HLA-II peptide signal approximately proportional to the input amount analyzed (Supplementary Fig. S4). No saturation effect was observed at any of the higher cell number samples, which provided evidence of linear response of the assay within this cell range. Furthermore, analysis of a second IP of the supernatant following the first IP confirmed that the HLA alpha chain recovery was typically > 95% and ≥ 90% in all conditions. This included cell lysate equivalents up to 100,000 stimulated DCs with increased HLA-II levels for at least three sets of DCs (Supplementary Fig. S5). Therefore, and in the absence of HLAII α-chain protein reference standards, the concentrations determined from this procedure were considered a suitable quantitative measure of HLAII alpha chain abundance.

### HLA-II α-chain abundance in cell lines by LC–MS/MS

**Figure 5:**
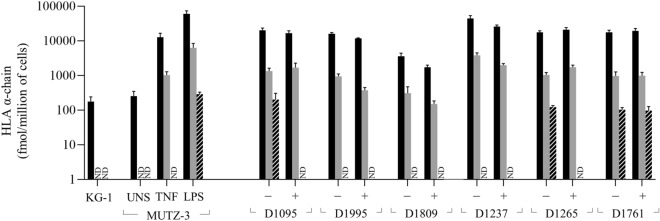
Amount of HLAII α-protein per million dendritic-like cells and DCs before (−) and after (+) LPS activation. Quantification of endogenous HLA-DRA1/DQA2 (Black bars), -DPA1 (Gray bars) and -DQA1 (Striped bars) based on the normalization with the signal of stable isotope labeled (SIL) peptides. Total amount of each HLA chain was back calculated by multiplying the normalized endogenous signal by the amount of SIL used. Each bar represents the mean and the SD of three samples corresponding to 25,000, 50,000 and 100,000 cells, each of them injected in duplicate and previously averaged. UNS, Unstimulated cells; ND, Peptide not detected.

For KG-1 cells, only the HLA-DRA1 derived peptide was detected, at 176 fmol/1 × 10^6^ cells, while DPA1 and DQA1 peptides were undetectable (Fig. 5). Because the fundamental antigen presentation pathways are misregulated in KG-1, we then examined HLA-II levels in both unstimulated and stimulated MUTZ-3 cells. While HLA-DRA1 was detected at 268 fmol per million unstimulated MUTZ-3 cells, like in KG-1 cells, HLA-DPA1 and -DQA1 proteotypic peptides were also undetectable in unstimulated MUTZ-3 (Fig. 5). MUTZ-3 cells induced with TNF and LPS showed a significant elevation of HLA-DRA1/DQA2-levels corresponding to 12 and 60 pmol per million cells, respectively, representing a 50- and 200-fold increase over the unstimulated condition (Fig. 5). However, HLA-DPA1 peptide was also upregulated in MUTZ-3 cells and became detectable at 2.2 and 6.2 pmol per million cells with TNF- and LPS-stimuli, respectively, representing between 7.5 and 9.4% of the total HLA-II amount (Fig. 5). Notably, HLA-DQA1 peptide was detected only after LPS-maturation signal at 288 fmol per million cells, being the lowest HLA α-chain detected in MUTZ-3 (Fig. 5), constituting < 1% of the total HLAII detected.

The quantitative HLA-II α-chain LC–MS/MS method, in comparison to the WB method, allowed for a more reliable and specific assessment of total HLA-DRA1 expression, which increased 50- and 200-fold in MUTZ-3 in response to LPS and TNF stimulation, respectively. In contrast, csHLADR levels measured by flow cytometry increased only 3- to 4-fold, suggesting that only a fraction of the newly synthesized HLA-II protein is translocated to the cell surface upon LPS and TNF stimuli.

### HLA-II α-chain abundance in DCs by LC–MS/MS

We next analyzed HLA-α chain levels in human monocyte-derived DCs, where HLA-DRA1 was the most abundant variant detected in the six sets of donor-derived DCs, corresponding to ≥ 90% of total α-chain (Fig. 5). Quantification of HLA-DRA1 showed some variability between donors, resulting in 20.2, 16.0, 3.7, 44.1, 17.5 and 17.7 pmol/1 × 10^6^ unstimulated DCs for D1095, D1995, D18092, D1237, D1265, D1761, respectively. HLA-DPA1 was the second most abundant α-chain in all cases, representing 5 to 10% of total HLA-α chain at 1.3, 0.9, 0.3, 3.8, 1.0 and 0.9 pmol/1 × 10^6^ donor-derived cells from of D1095, D1995, D18092, D1237, D1265, D1761, respectively (Fig. 5). In contrast, HLA-DQA1 was detected only in three donors (D1095, D1265, D1761) at very low levels (~ 0.1 pmol/1 × 10^6^ of DCs), constituting < 1% of total α-chain when detected (Fig. 5). Of note, loss of DQA1 expression in D1095 and D1265 was observed after LPS stimulation.

**Figure 6:**
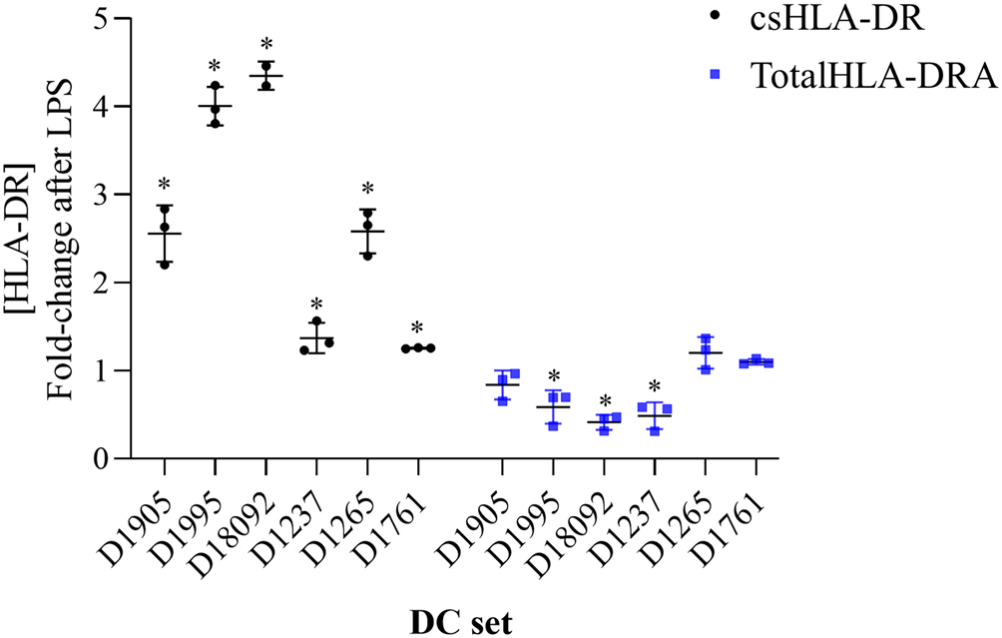
DCs display increased levels of csHLA-DR upon LPS-activation in contrast with total levels HLA-DRA1 α-chain. csHLA-DR and totalHLA-DRA1 fold induction between unstimulated and LPS-treated DCs, statistical significance was analyzed with a two-tailed Student's *t* Test (*p < 0.05). Each point for csHLA-DR represents a replicate (n = 3). For totalHLA-DRA1 each point represents the mean for two injections corresponding to 25,000, 50,000 and 100,000 cells per donor (n = 3) and the SD (error bars).

While morphologic and immunophenotypic changes were observed in all LPS-treated DCs, the ratio of abundance of HLA-DRA1, -DPA1 and -DQA1 proteins remained relatively consistent before and after LPS induction (> 90%, 5–10% and < 1%, respectively). Three sets of LPS-stimulated DCs (D1995, D1809 and D1237) showed a significant decrease in total amount of HLA α-chain detected compared with unstimulated cells (30–70% reduction). In contrast, HLA-II levels in the remaining sets of DCs (D1905, 1237 and 1761) did not change significantly after LPS treatment. These results contrast with the increase in HLA-II detected in the cell line model MUTZ-3, where total HLAII increased upon stimulation. The variable response to LPS activation in donor-derived DCs may be due to factors such as HLA background of donors, as well as multifactorial regulation of the molecular players in the biosynthesis of the HLA polypeptides and its translocation mechanisms from the intracellular compartment to the cell surface in response to maturation signals. While acute myeloid leukemia-derived cell lines KG-1 and MUTZ-3 exhibited low levels of cell surface HLA-DR, suggesting the low abundance of HLA-II and resembling their lineage origin as CD34+ DC-precursors, HLA-II quantification in primary donor-derived DCs by MS demonstrated a decrease or no change in total levels of HLA-DRA1, -DPA1 and -DQA1 after LPS induction, which contrasts with the increase of HLA-DR at the cell membrane observed in all cases (Fig. 6). Altogether these results indicate a differential response of HLA-II levels to the activator LPS, which may respond to the individual variability associated with haplotype and other factors involved in the biosynthesis, translocation and stabilization of HLA-II at the cell membrane.

## Conclusions

The elucidation of total HLA-II levels of represents a valuable opportunity to build our understanding of antigen presentation and subsequent T-cell activation. Here we present the first study that elucidates the abundance of the total levels of HLA-II protein by targeting specifically α-chain polypeptides, which ranged from 100 to 200 fmol per million dendritic-like cells and 3–30 pmol per million unstimulated and stimulated DCs. These amounts correspond to 6–12 × 10^4^ and 1.8 × 10^6^–1.8 × 10^7^ HLA-II molecules per cell for cell lines and DCs, respectively. Consistent with a previous published results^34–41^, we confirmed an increase of csHLADR following TNF and LPS treatment for the monocytic-like cell line MUTZ-3, where both stimulants elicited a response of similar magnitude. This is also consistent with the increase in allogenic T cell-activation potential by induced-MUTZ-3 found by T cell proliferation assays^38^. However, our assessment of total HLA-II abundance by mass spectrometry showed a larger effect in stimulated MUTZ-3 cells, suggesting that an accumulation of HLA- α chain may occur at the intracellular compartments. Moreover, the varied response to both proinflammatory stimuli could be explained by the high heterogeneity in MUTZ-3 cells despite the clonal origin^53–55^, . Furthermore, signal transduction upon different maturation signals may be also affected by cell cycle phase and receptor-mediated responses at the single-cell level. Overall, these findings are consistent with the proinflammatory response by MUTZ-3 differentiated into DCs in cytokine production and chemokines as well as the immunophenotypic profile reported, which maturation more closely recapitulates that of DCs^34–36,38,41^.We have shown that HLA-DRA1 protein is the most abundant α-chain variant (> 90%), followed by -DPA1 (3–7%) and -DQA1 (< 1%) when detected, irrespective of the HLA-II haplotype in dendritic-cell lines KG-1 and MUTZ-3. This was also observed in monocyte-derived DCs, where HLA-DRA1 levels were consistently higher than -DPA1, and -DPA1 was consistently higher than DQA1. These findings are consistent with the predominance of HLA-DRA1 followed by -DPA1 and -DQA1 recently reported at the transcriptional level by RNA-seq^49^. In half the donor samples DQA1 was not detected. In the case of KG-1 cells DPA1 and DQA1 were not detected by MS despite the haplotype (DRB1*11:01:02, *14:01:01G, DRB302:02:01G DQA1*01:01,*05:01; DPA1*02:01 and *03:01). Furthermore, because the equivalent relative abundance of HLA-DRA1 and -DQA1 at the transcriptional and translational level have been previously reported^10,11^, it is possible to hypothesize that differential mechanisms of the protein synthesis and half-life underline HLA-II abundance.

Noteworthy, HLA-DQA1 levels were downregulated after LPS induction in two sets of DCs (D1095 and D176), suggesting differential stability mechanisms for this variant. For example, while MARCH1 and MARCH8 E3 ligases are known to ubiquitinate HLA-II molecules in order to maintain them in the endosomal and lysosomal compartments for storage and degradation^23–25,56–59^, HLA-DQ has been found ubiquitinated by an alternative ligase (MARCH9)^58,59^. Furthermore, monocytic differentiation into DCs and maturation induced by GM-CFS+ IL-4 and LPS, respectively led to a signaling cascade of upstream activators in a rapid manner^7,60–64^, which leads to a transient increase in de novo MHCII synthesis at the transcriptional level, followed by a subsequent decrease resulting from silencing from its transcriptional activator CIITA^14,29,30^. Therefore, potential differences in the rates of CIITA silencing and transcriptional shutdown in DCs at the time of harvest and analysis could impact the quantification depending on the individual variability. For example, the detection of HLA-DRA1 and -DQA1 transcripts at the same levels after 6 h of stimulation with LPS changed significantly for -DQA1, where its expression dropped to baseline levels after 24 h^10^. Thus, differences in the developmental state and differentiation intermediaries when monocyte-derived dendritic cells were stimulated with LPS and harvested at 22 h cannot be ruled out**.** Furthermore, the low abundance of HLA-DQA1 highlights a fundamental question regarding its association with a wide range of human disorders such as celiac disease and the potential role of HLA-DQA1 protein levels change in disease processes.

While the focus of this work is on method development for MS quantification of HLA-II, further application of this method will complement characterization studies of DC maturation and other processes relevant to DC biology. Thus, quantification of total HLA α-chains is expected to contribute to key research fields in the context of adaptative immunity such as immunopeptidomics, transplantation and vaccine studies. Finally, because HLA abundance differences appear to be individual-, cell- and haplotype-specific markers in cancer^65–69^, MS quantification of HLA α-chains may contribute to use of HLA-II as a potential clinical biomarker.

## Supplementary information

Supplementary information.

## Data Availability

The datasets generated and analyzed during the current study are available from the corresponding author upon request.
