# Supplementary material for: Human leukocyte antigen class II quantification by targeted mass spectrometry in dendritic-like cell lines and monocyte-derived dendritic cells
Source: Sci Rep. 2021 Jan 13;11:1028. doi: 10.1038/s41598-020-77024-y (PMC7807004; doi:10.1038/s41598-020-77024-y)
Supplement: Supplementary file 1 — Supplementary information. [file 41598_2020_77024_MOESM1_ESM.pdf]

Human Leukocyte Antigen class II quantification by targeted mass spectrometry in dendritic-like cell lines and monocyte-derived dendritic cells

Casasola-LaMacchia A[1*], Ritorto MS[1], Seward RJ[1], Ahyi-Amendah N[2], Ciarla A[2], Hickling TP[2] and Neubert H[1]

[1]Quantitative Biomarkers & Biomeasures, BioMedicine Design, Pfizer, Inc., Andover, MA, USA. [2]Immunogenicity Sciences, BioMedicine Design, Pfizer, Inc., Andover, MA, USA.

**e-mail:* Marcela.Casasola-LaMacchia@pfizer.com

Western blot. To obtain whole lysates, pelleted cells were thawed and resuspended in 1 mL of cold lysis buffer (20 mM Tris ,150 mM NaCl, 1% v/v CHAPS + 1 mM PMSF (Sigma, A3428-10MG), 5 µg/mL Aprotinin (Sigma, A3428-10MG), Pepstatin A 10 µg/mL (Calbiochem, 516481-5MG) and Leupeptin 10 µg/mL (Sigma, L5793-5MG)). Lysates were incubated for 1 h with end-to-end rotation at 4°C. Lysates were pelleted at 2,500 g for 5 min and the supernatant was recovered. Protein concentration was measured using a BCA assay kit (Pierce, 23238). 10 µg of protein was resuspended in SDS loading buffer (Novex life technologies, N00007), denatured at 70C for 10 min and resolved on a 4–12% acrylamide gel (Invitrogen Life technology, NP0321BOX), at 100 Volts for 1 h. The proteins were transferred to a PVDF membrane (Life technologies, 88518) in 20% Methanol TRIS-Glycine-SDS buffer at 100 Volts for 1 h. After transfer, the membranes were blocked in LI-COR blocking buffer (LI-COR Odyssey, 927–40000) overnight at 4°C and then blotted with the primary antibodies for 2h at RT. The membranes were washed 3 times with TBST (0.1 % Tween 20, Tris-buffered saline, TBST) then incubated with secondary antibodies in blocking buffer for 1h at RT. Membranes were then washed and the signal was detected by a Cx LI-COR Odyssey infrared imaging system (LI-COR Biosciences). The infrared imaging system was used to detect HLA-DR levels as ratios with the endogenous loading control β-actin. Three normalized ratios of 2 biological replicates were averaged and P values were obtained with a two-tailed Student's t-Test (heteroscedastic). Primary antibodies used were mouse anti-HLADR (1:1000 dilution; TAL 1B5 \Thermo Fisher Scientific, MA1 46109) and rabbit anti-β-actin (1:1000, Novus, NB100-56874). Secondary antibodies used were donkey anti-rabbit IRDYE 680CW and anti-mouse IRDYE 800CW (1:15000 dilution; LI-COR Biosciences, 926–68073 and 926–32212) antibodies.

```
sp|P01906|DQA2_HUMAN      ------MILNKALLLGALALTAVMSPCGGEDIVADHVASYGVNFYQSHGPSGQYTHEFDG
sp|P01909|DQA1_HUMAN      ------MILNKALMLGALALTTVMSPCGGEDIVADHVASYGVNLYQSYGPSGQYTHEFDG
sp|P01903|DRA_HUMAN       ------MAISGVPVLGFFIIAVLMSAQESWAIKEEHVIIQ-AEFYLNPDQSGEFMFDFDG
sp|P20036|DPA1_HUMAN      MRPEDRMFHIRAVILRALSLAFLLSLRGAGAIKADHVSTY-AAFVQTHRPTGEFMFEFDE
                                *    . :*  : :: ::*   .  *  :**    . :  .   :*:: .:**

sp|P01906|DQA2_HUMAN      DEEFYVDLETKETVWQLPMFSKFISFDPQSALRNMAVGKHTLEFMMRQSNSTAATNEVPE
sp|P01909|DQA1_HUMAN      DEQFYVDLGRKETVWCLPVLRQFR-FDPQFALTNIAVLKHNLNSLIKRSNSTAATNEVPE
sp|P01903|DRA_HUMAN       DEIFHVDMAKKETVWRLEEFGRFASFEAQGALANIAVDKANLEIMTKRSNYTPITNVPPE
sp|P20036|DPA1_HUMAN      DEMFYVDLDKKETVWHLEEFGQAFSFEAQGGLANIAILNNNLNTLIQRSNHTQATNDPPE
                          ** *:**:  ***** *  : :   *:.* .* *:*: : .*: : ::** *  **  **

sp|P01906|DQA2_HUMAN      VTVFSKFPVTLGQPNTLICLVDNIFPPVVNITWLSNGHSVTEGVSETSFLSKSDHSFFKI
sp|P01909|DQA1_HUMAN      VTVFSKSPVTLGQPNILICLVDNIFPPVVNITWLSNGHSVTEGVSETSFLSKSDHSFFKI
sp|P01903|DRA_HUMAN       VTVLTNSPVELREPNVLICFIDKFTPPVVNVTWLRNGKPVTTGVSETVFLPREDHLFRKF
sp|P20036|DPA1_HUMAN      VTVFPKEPVELGQPNTLICHIDKFFPPVLNVTWLCNGELVTEGVAESLFLPRTDYSFHKF
                          ***:.: ** * :** *** :*:: ***:*:*** **. ** **:*: **.: *: * *:

sp|P01906|DQA2_HUMAN      SYLTFLPSADEIYDCKVEHWGLDEPLLKHWEPEIPAPMSELTETLVCALGLSVGLMGIVV
sp|P01909|DQA1_HUMAN      SYLTLLPSAEESYDCKVEHWGLDKPLLKHWEPEIPAPMSELTETVVCALGLSVGLVGIVV
sp|P01903|DRA_HUMAN       HYLPFLPSTEDVYDCRVEHWGLDEPLLKHWEFDAPSPLPETTENVVCALGLTVGLVGIII
sp|P20036|DPA1_HUMAN      HYLTFVPSAEDFYDCRVEHWGLDQPLLKHWEAQEPIQMPETTETVLCALGLVLGLVGIIV
                           **.::**::: ***:*******:******* : *  :.* **.::***** :**:**::

sp|P01906|DQA2_HUMAN      GTVFIIQGLRSVGASRHQGLL
sp|P01909|DQA1_HUMAN      GTVFIIRGLRSVGASRHQGPL
sp|P01903|DRA_HUMAN       GTIFIIKGVRKSNAAERRGPL
sp|P20036|DPA1_HUMAN      GTVLIIKSLRSGHDPRAQGTL
                          **::**:.:*.   .. :* *
```

Supplementary Figure S1. Multiple sequence alignment of HLA-DRA1/DQA2, -DPA1 and -DQA1 proteins (Clustal 2.1). Proteotypic peptides utilized for quantification by MS are highlighted.

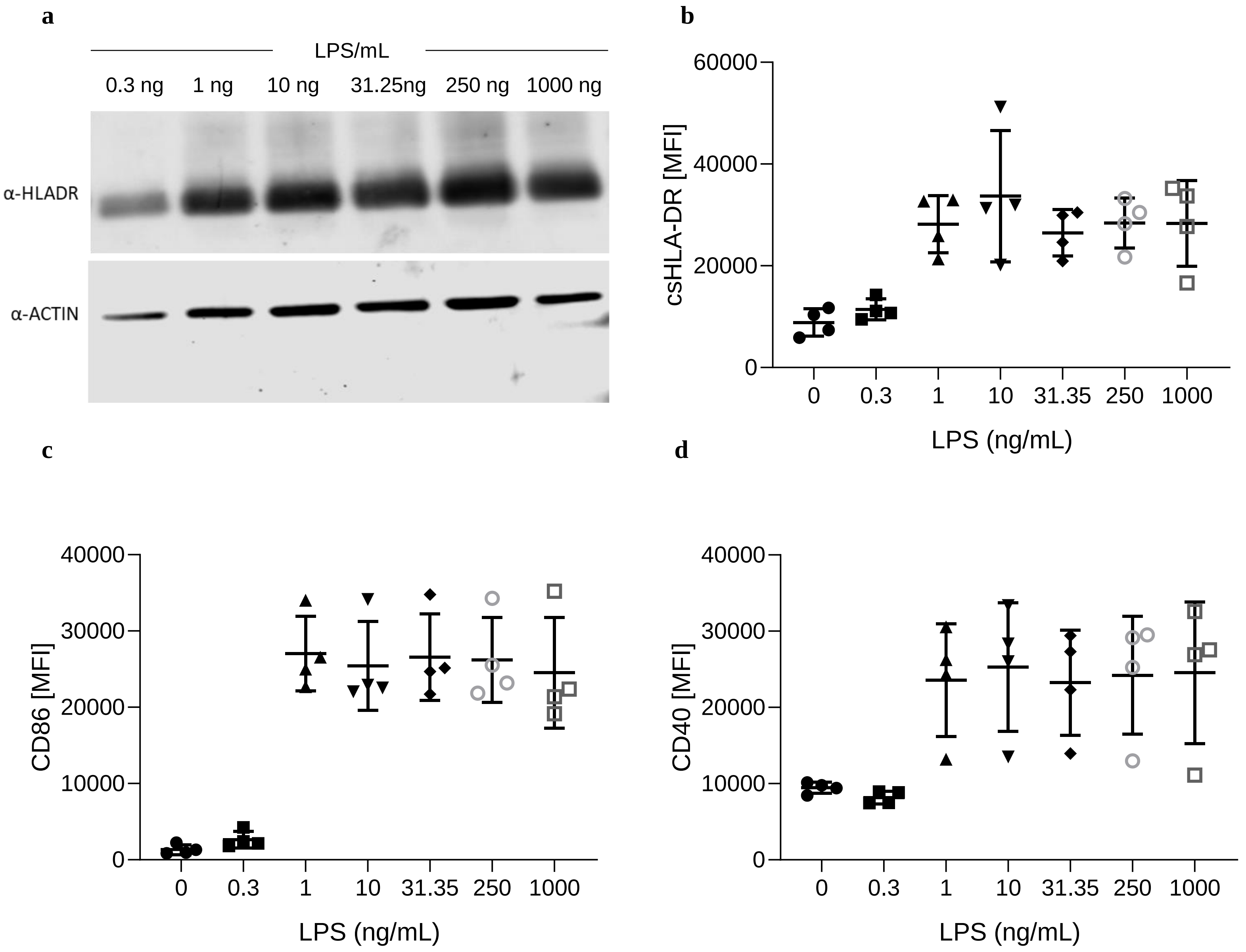

Supplementary Figure S2. MUTZ-3 stimulation with different doses of LPS. (A) HLA-DR detected by western blot in DCs treated independently with different doses of LPS (n=4), actin was used as a loading control, a representative set is presented. (B-C) csHLA-DR-FITCI, CD40-APC and CD-86-PE median fluorescence intensity (MFI) is presented in the y-axis per marker. Each data point represents a replicate per donor sample (n=4), and the respective error bars (SD).

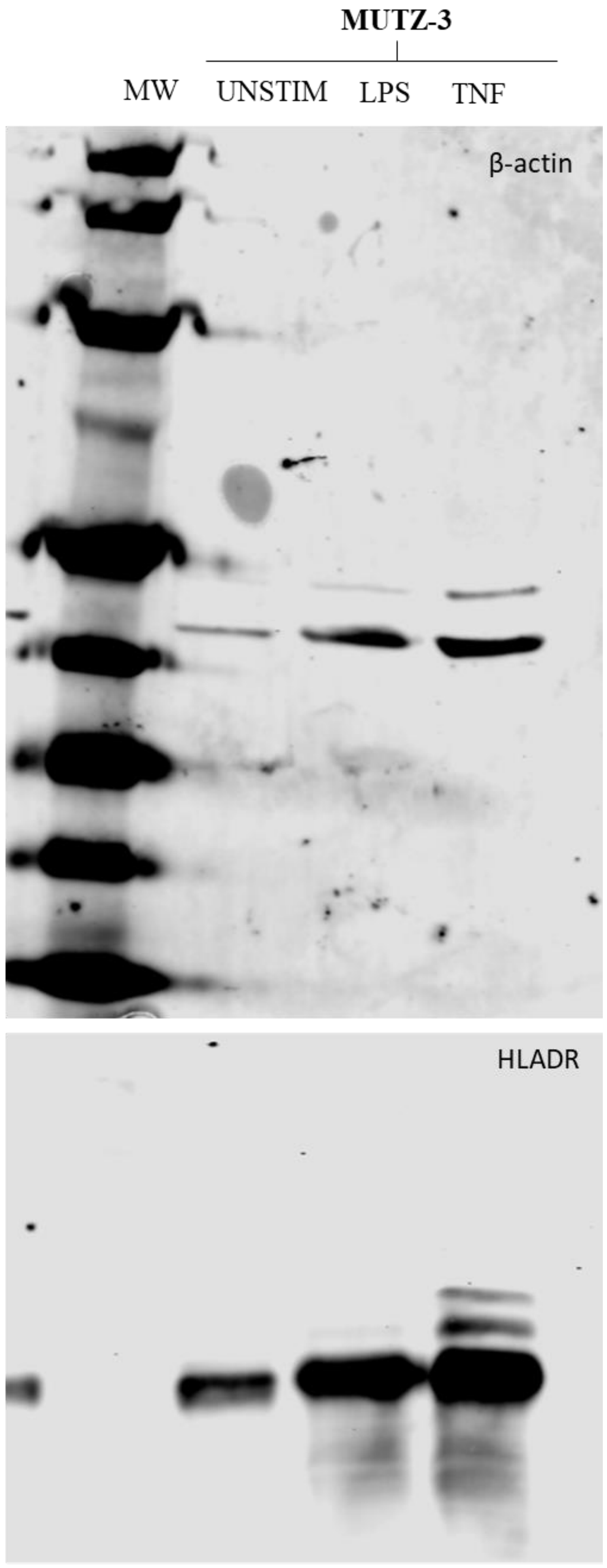

Supplementary Figure S3. Detection of HLA-DR and β -actin protein and in whole-cell lysates from DCs MUTZ-3 cells by western blot. Complete blot with molecular weight ladder (MW) is presented, where the same PDVF membrane with the loaded lysates was blotted with the antibody TAL-1B5 (Top panel) and rabbit anti-β-actin (Bottom panel) for further detection with the secondary antibodies as the method described above.

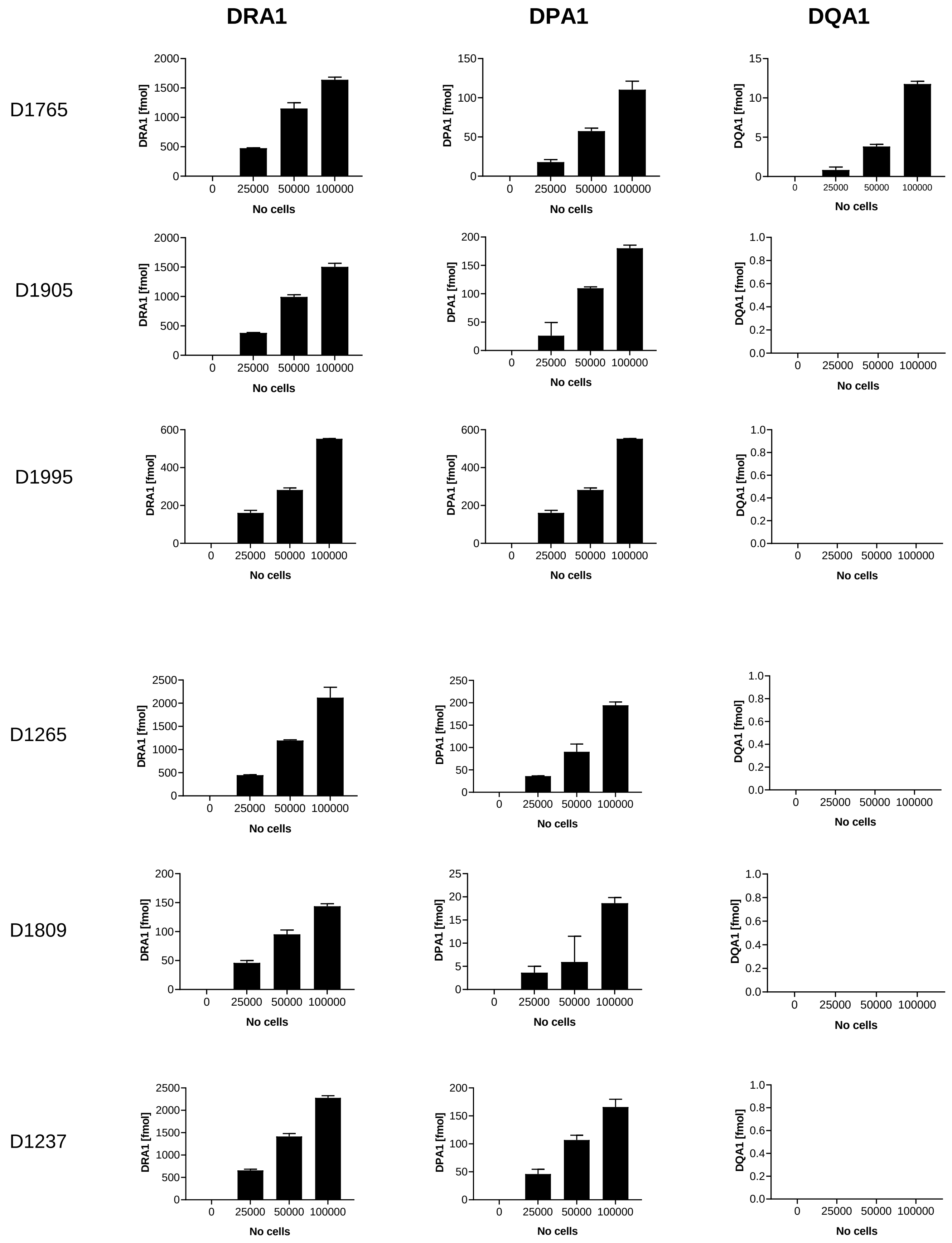

Supplementary Figure S4. MS-based titration curves of detection of HLA-α chains in whole-cell lysates from monocity DCs. Total amount of each HLA chain is shown (x-axis), which was back calculated by multiplying the normalized endogenous signal by the amount of SIL used. Each bar represents the mean and standard deviation (error bars) of two samples corresponding to 25,000, 50,000 and 100,000 cells.

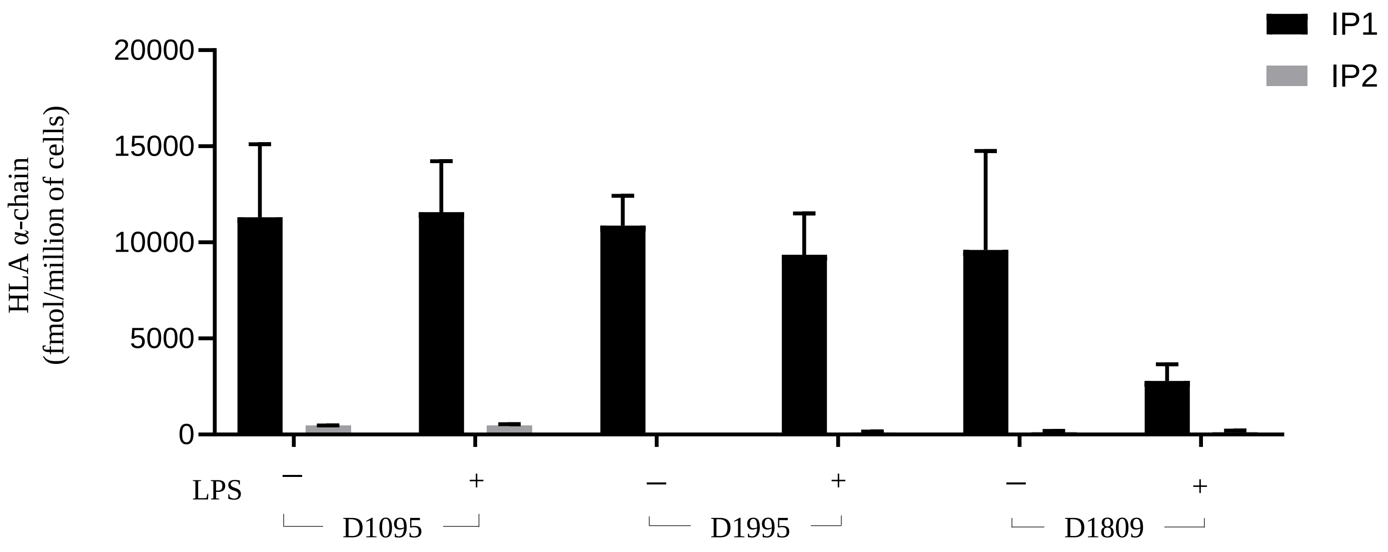

Supplementary Figure S5. Recovery of HLA-α chains in whole-cell lysates from monocity DCs upon two sequential immunoprecipitations (IP). Total amount of endogenous HLA chains detected by MS after IP1 and IP2 was calculated. Each bar represents the mean of three samples corresponding to 25,000, 50,000 and 100,000 cells and the respective SD (error bars).

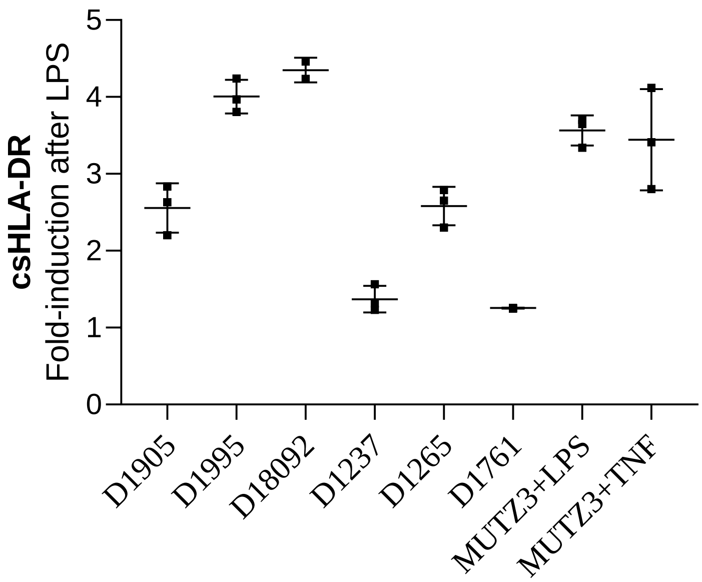

Supplementary Figure S6. csHLA-DR upon maturation in DCs and MUTZ-3 cells. csHLA-DR fold induction is presented (x-axis). Each data point represents a replicate for a n=3 per sample and the respective SD. Statistical significance was evaluated with a paired two-tailed Student's t-test, where a significant difference was found for all cases ($p<0.05$).
